# Spatial differentiation and geographical similarity of traditional villages——Take the Yellow River Basin and the Yangtze River Basin as examples

**DOI:** 10.1371/journal.pone.0295854

**Published:** 2024-02-02

**Authors:** Zhongyi Nie, Tian Dong, Wei Pan

**Affiliations:** School of Architecture and Art, Central South University, Changsha, China; East China Normal University, CHINA

## Abstract

The preservation and advancement of Traditional Villages are intricately linked to the perpetuation of cultural heritage. However, existing research on the spatial disparities among Traditional Villages has yet to consider the impact of cultural factors. Moreover, the geographical similarities shared by Traditional Villages have not been thoroughly examined. Therefore, this study takes the Yellow River Basin and the Yangtze River Basin, both pivotal in the genesis of Chinese culture, as case studies. We employ an Optimal Parameter-based GeoDetector alongside the Spatially Constrained Multivariate Clustering method to delve into the factors influencing the spatial differentiation and geographical similarities of Traditional Villages within these two significant river basins. Subsequently, we offer recommendations for fostering the sustainable preservation and development of Traditional Villages in these regions. The results indicate that the Rural Residents Per Capita Disposable Income has the greatest impact on the spatial differentiation of Traditional Villages in the Yellow River Basin, while the Density of National Intangible Cultural Heritage Inheritors has the most significant influence in the Yangtze River Basin. The interaction effects of the driving factors are more powerful, with a q-value of 0.9544 for the interplay between the Density of National Cultural Protection Units and the Tourism Income in the Yellow River Basin and a value of 0.9099 for the interaction between the Density of National Intangible Cultural Heritage Inheritors and the Transportation in the Yangtze River Basin. Regarding geographical similarity, the Traditional Villages in the Yellow River Basin are divided into three major clusters, while those in the Yangtze River Basin are divided into two.

## Introduction

Traditional Villages (TVs), as a tangible and intangible heritage of agricultural civilizations, embody the vitality of world agrarian civilizations. They are the nurturing grounds and bearers of ethnic cultures, recording the traces of historical events and the advancement of society. They serve as the genetic code of human civilization, providing significant clues for uncovering the historical and cultural contexts of different eras and regions [1–3]. However, the problem of rural decline has emerged as a fundamental issue stemming from global urbanization and industrialization [4]. Extensive research has already provided evidence of rural decline in developed countries such as Europe, America, and China [5–7]. According to the Chinese National Bureau of Statistics, the number of villages in China was 3,773,000 in 1990 and 2,633,000 at the end of 2021, a decrease of 1,140,000 in 31 years, with a mean of 101 villages going away daily, and relevant studies have shown that the hollow index of Chinese Traditional villages (CTVs) has exceeded 0.5 [8]. Moreover, according to statistics published by Chinese National Bureau of Statistics, by the end of 2022, China’s urbanization rate has reached 65.22%, which will mean that nearly 300 million people will still be converted from rural to urban household registration in China in the next 30 years, according to the standard of 80% to 90% urbanization rate in developed countries, which show that TVs in China are facing a variety of issues, notably "hollowing out", urban development, aging of traditional buildings, resource development, excessive tourism, and ecological pollution, and the protection of TVs is a matter of urgency [9–14].

As early as the 1840s, the German geographer J.G. Cole researched the classification and development model of settlements [15]. Subsequently, esteemed scholars such as Paul, Albert, Jean, and others began extensive research to delve deeper into the morphology, functions, distribution, and diverse geographical phenomena related to rural settlements [16–18]. In recent years, the study of TVs has gained significant attention in academic disciplines such as architecture, geography, tourism, urban planning, and related fields [10, 19–21]. Numerous scholars have conducted extensive case studies and combined theoretical analysis on various aspects of TVs, including rural landscapes [22, 23], spatial reconstruction [24], spatial morphology [1, 25], architectural dwellings [26], and spatial distribution [27, 28]. However, the consideration of issues affecting the spatial configurations of TVs is not sufficiently in-depth. Most studies primarily pay attention to the influences of the natural environment, culture, and economy, with limited attention to cultural factors [29, 30]. They only consider the impact of cultural heritage and overlook the effects of factors closely related to TVs, such as genealogy and language. Furthermore, the consideration of economic factors is limited to primary, secondary industries and per capita GDP [27], without considering factors that directly indicate the state of rural development, like per capita disposable income and tourism revenue. Moreover, existing research have not explored the geographic similarities among TVs. Additionally, no studies have investigated the space pattern of TVs in the Yangtze River Basin (YZRB). The Yellow River Basin (YLRB) and the YZRB, both regarded as the birthplaces of Chinese civilization, have not only fostered a rich tapestry of diverse ethnic cultures but have also engendered intricate human-environment dynamics [31]. These areas have not merely upheld and perpetuated the legacy of Chinese culture throughout history but have also played a pivotal role in safeguarding ecological integrity and advancing the economic development of the Chinese nation [29]. Therefore, conducting a comprehensive exploration of the influencing factors responsible for the spatial distribution of TVs within these two significant river basins, along with a deeper investigation into the geographical commonalities among these villages, stands to significantly contribute to the development of well-grounded strategies for the preservation and advancement of TVs. Consequently, it will facilitate the enduring preservation and progression of Chinese culture.

This study aims to address several vital objectives to bridge the existing research gap. Firstly, it analyzes the spatial distribution and driving factors influencing TVs in the YLRB and YZRB. Secondly, it aims to explore the optimal categorization of these driving factors to evaluate their impact accurately. Lastly, it intends to investigate the variations in geographical similarities among TVs in the two basins. By considering a comprehensive set of 17 driving factors encompassing the natural environment, social culture, and space economy, this study endeavors to provide comprehensive insights into the aforementioned research areas.

## Materials and methods

### Study area

**Fig 1:**
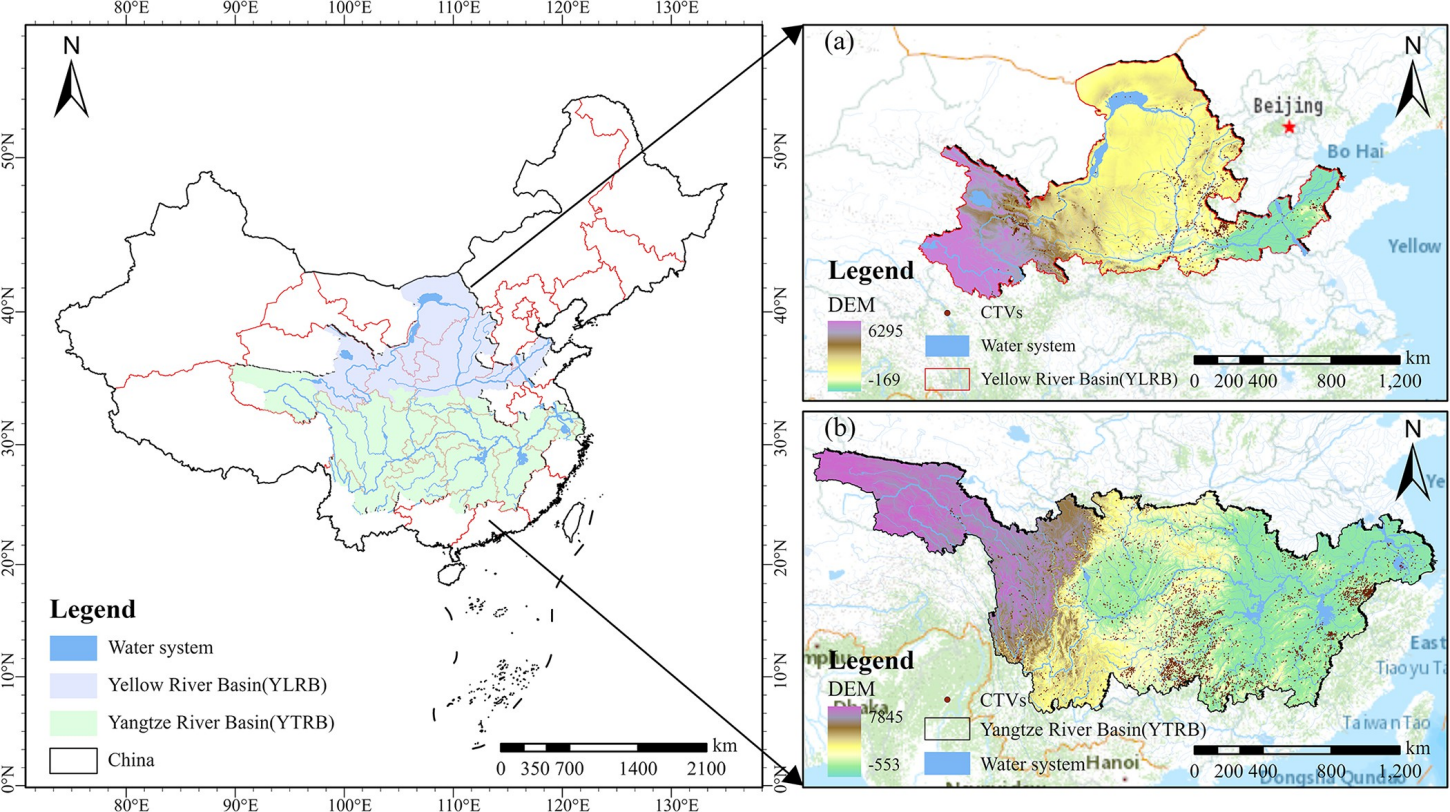
Study area. The maps were generated by ArcGIS 10.6 and were for illustrative purposes only.

The mother rivers of China are the Yangtze and Yellow rivers, which have basin areas of about 2.8 million square kilometers, 30% of China’s geographical area, and carry close to one billion people. They have fostered a magnificent national culture and are key hubs of China’s economic development. 7,000 years ago at the earliest, agricultural settlements were established here, leaving behind numerous rich heritages and traditional ways of life in ancient villages [32–34]. As of May 2023, there are 1,129 TVs in the YLRB and 3,690 in the YZRB. The YLRB and the YZRB have distinct geographical and ecological characteristics, with significant climate, topography, and vegetation differences (Fig 1). The two regions’ different historical, cultural, and economic backgrounds have resulted in their unique spatial distributions of TVs. In addition to advancing economic growth, the promotion of cultural variety is a crucial element in development since it improves individuals’ intellectual, emotional, ethical, and spiritual health [35, 36]. A deep understanding of the space pattern differences of TVs in the two basins and their influencing factors is crucial for protecting and promoting TVs and disseminating historical culture.

### Data sources

The data that was used in this investigation is shown in Table 1. The dependent variable is the distribution density of TVs, and the 17 independent variables are divided into three groups as follows: natural environment, social culture, and space economy. The Natural Environment (NE) category comprises Elevation (ELE), Relief (RE), Climate Zones (CZ), and Distance from the water system (DW); the Social Culture (SC) category comprises the Density of National Intangible Cultural Heritages (DoNICH), Density of National Intangible Cultural Heritage Inheritors (DoNICHI), Density of National Cultural Protection Units (DoNCPU), Number of Minorities (NoM), Number of Genealogies (NoG), Language Zones (LZ), Density of National A-class Tourist Attractions (DoNATA), and Density of National Parks of China (DoNPoA); the Space Economy (SE) category includes Transportation (TRA), Urbanization (URB), Population (POP), Tourism Income (TI), and Rural Residents Per Capita Disposable Income (RRPCDI).

**Table 1:** Summary of data sources.

Type		Application	Source	Resolution
Natural Environment	DEM	ELE, RE	Geospatial Data Cloud (https://www.gscloud.cn/search)	30m
	Climate Zones	CZ	Resource Environment and Data Center of the Chinese Academy of Sciences (https://www.resdc.cn/data.aspx?DATAID=124)	/
	Water system	DW	OpenStreetMap (http://www.openstreetmap.org/)	/
Social Culture	Cultural heritage	DoNICH, DoNICHI, DoNCPU	China Intangible Cultural Heritage Network (https://www.ihchina.cn/)	/
	Minorities	NoM	The 7th China Population Census	/
	Genealogies	NoG	General Catalogue of Chinese Genealogy	/
	Language	LZ	Atlas of Chinese Languages	/
	Scenic areas	DoNATA, DoNPoA	Chinese Ministry of Culture and Tourism (https://www.mct.gov.cn/)	/
Space Economy	Roads	TRA	OpenStreetMap (http://www.openstreetmap.org/)	/
	City government location	URB	Baidu map (https://map.baidu.com/@12576415,3251499,13z)	/
	Population	POP	LandScan Global Population Data2021 (https://landscan.ornl.gov/)	1km
	Income	TI, RRPCDI	China City Statistical Yearbook	/

## Methods

**Fig 2:**
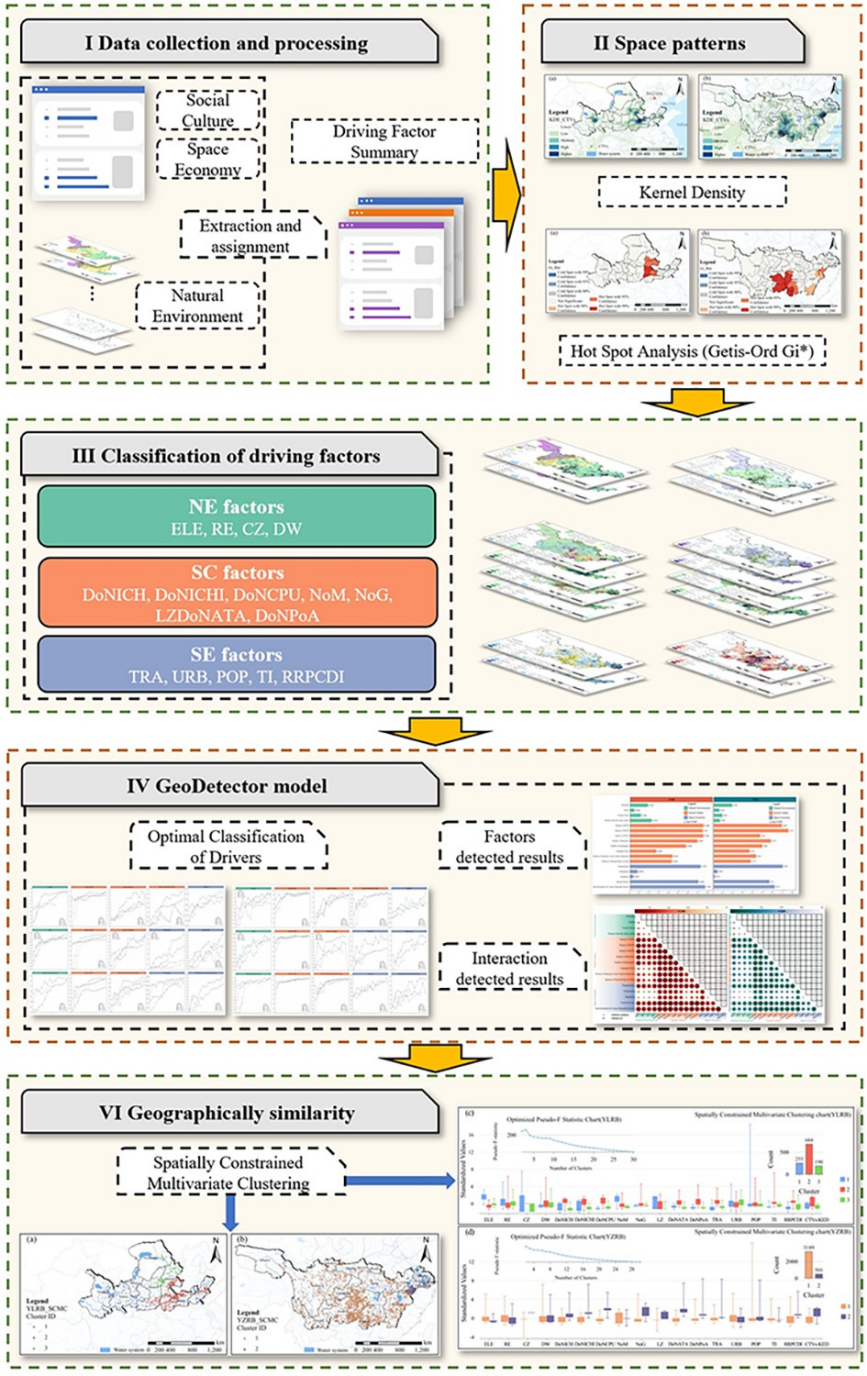
Technical route. The maps were generated by ArcGIS 10.6 and were for illustrative purposes only.

This study consists of five steps (Fig 2). The first step involves data collection and processing, followed by using ArcGIS to analyze the spatial configuration of TVs in YLRB and YZRB and to perform statistical classification of various driving factors. In the third stage of this study, a comparative statistical analysis was carried out to investigate the differences in the distributional impacts of different driving elements on TVs in YLRB and YZRB. In the fourth stage, the attribute values of various driving factors were integrated into the Optimal Parameter-based GeoDetector (OPbGD) to obtain the optimal classification with the maximum q value to investigate spatial heterogeneity and assess driving factors’ maximum degree of influence. The obtained data were used to analyze the interaction mechanisms among various factors. Finally, Spatially Constrained Multivariate Clustering (SCMC) was employed to perform geographical similarity analysis and obtain the optimal classification that maximizes intra-class similarity and inter-class differences in the regional scale of CTVs.

### Multi-distance spatial cluster analysis

Ripley’s K-function is a widely used method for exploring the spatial distribution configurations of point elements at arbitrary scales [37, 38]. It is commonly employed to analyze the distribution of point features, such as TVs. The formula for calculating Ripley’s K-function is shown in Eq (1):

$$M(d)=\sqrt{\frac{C\sum_{a=1}^{m}\sum_{b=1,a\neq b}^{m}k_{a,b}}{\pi m(m-1)}}$$ (1)

Where m is the number of TVs in the study area, d is the distance, and C is the area of the study area. *k*_*a*,*b*_ is the weight, when the distance between a and b is less than d, *k*_*a*,*b*_ = 1; when the distance between a and b is greater than d, *k*_*a*,*b*_ = 0.. If *M*(d) is greater than 0, it indicates that the TVs exhibit a clustered distribution, while *M*(d) less than 0 suggests a dispersed distribution.

### Kernel density analysis

The kernel density (KDE) analysis method illustrates alterations in the space pattern of point features at the regional scale and indicates the cohesion level among TVs [39, 40]. The formula below demonstrates this approach:

$$E(z)=\frac{1}{md}\sum_{a=1}^{m}k(\frac{z-z_{a}}{d})$$ (2)

In Eq (2), E(*z*) is the KDE estimate with *d* as the search bandwidth. Additionally, *m* represents the count of point elements, (*z*−*z*_*a*_) denotes the distance from the estimated point *z* to *z*_*a*_, $k(\frac{z-z_{a}}{d})$ is the kernel function.

### Local spatial correlation

In 1970, Tobler introduced the First Law of Geography, which asserts that everything is related to everything else, but geographically closer objects have a stronger connection than those farther apart [41]. The Getis-Ord Gi* is a widely used method to detect local space autocorrelation, which provides a more precise identification of aggregation regions [42–44]. It is calculated using the following formula:

$$Gi*=\frac{\sum_{b=1}^{n}w_{a,b}x_{b}-\bar{x}\sum_{b=1}^{n}w_{a,b}}{m_{s}\sqrt{\frac{\left[n\sum_{b=1}^{n}w_{a,b}^{2}-{\left(\sum_{b=1}^{n}w_{a,b}\right)}^{2}\right]}{n-1}}}$$ (3)

In Eq (3), *x*_*b*_ is the attribute value of element b, *w*_*a*,*b*_ is the space weight between element a and element b, n is the total count of elements, $\bar{x}$ is the mean value, and *m*_*s*_ is the standard deviation of *x*_*b*_. Higher z-scores indicate the presence of significant space clustering of high attribute values, often referred to as hot spots, while lower z-scores indicate the presence of significant space clustering of low attribute values, often referred to as cold spots.

### Optimal parameter-based GeoDetector

GeoDetector is a commonly used model to recognize space heterogeneity and its related forces [45, 46]. The critical step in using GeoDetector is discretizing and classifying various driving factors into the optimal scale. The effectiveness of discretization classification is represented by q, where a larger q indicates a better classification effect [47, 48]. With the help of the GeoDetector package in R language (https://cran.r-project.org/web/packages/GD/vignettes/GD.html), equal interval classification, natural break classification, quantile classification, and geometric interval classification are used to set classification levels from 3 to 15. The maximum q is selected as the optimal parameter classification of spatial data. The OPbGD calculating formula is as follows:

$$q=1-\frac{\sum_{j=1}^{L}N_{j}\sigma_{j}^{2}}{N\sigma^{2}}$$ (4)

In the formula, j represents the stratification of variable A or element B. *N*_*j*_ and *N*, respectively, are the count of cells in stratum j and the overall region; $\sigma_{j}^{2}$ and *σ*^2^ represent the variance of A values in stratum j and area as the whole. The range of values for q is [0, 1], with higher values demonstrating greater spatial heterogeneity in attribute A. Higher values of q show a more prominent ability of the independent variable B to explain characteristic A when the independent variable B is the one that causes stratification.

### Spatially constrained multivariate clustering

Spatially Constrained Multivariate Clustering (SCMC) is a commonly used analytical method that considers both attribute values and positional geographic information [49]. It uses the SKATER approach and a connectivity graph (minimum spanning tree) to find naturally occurring clusters in the data and assemble evidence to determine the likelihood of cluster membership [50]. Additionally, it uses unsupervised machine learning. The Calinski-Harabasz pseudo-F-statistic, which measures the variance ratio between clusters to variance inside clusters, is used to assess the effectiveness of clustering. The formula for calculating F is:

$$\begin{array}{l} F=\frac{\left(\frac{Z^{2}}{m_{c}-1}\right)}{\left(\frac{1-Z^{2}}{m-m_{c}}\right)}\\ Z^{2}=\frac{SST-SSE}{SST}\\ SST={\sum_{a=1}^{m_{c}}\sum_{b=1}^{m_{a}}\sum_{r=1}^{m_{v}}\left(V_{ab}^{r}-\bar{V^{r}}\right)}^{2}\\ SSE={\sum_{a=1}^{m_{c}}\sum_{b=1}^{m_{a}}\sum_{r=1}^{m_{v}}\left(V_{ab}^{r}-\bar{V_{a}^{r}}\right)}^{2} \end{array}$$ (5)

Where m is the count of elements, *m*_*a*_ is the count of elements in cluster a, *m*_*c*_ is the count of clusters, *m*_*v*_ is the count of variables used to cluster elements, *V*_*ab*_^*r*^ is the value of the rth variable of the bth element in the ath cluster, $\bar{V^{r}}$ is the average value of the rth variable, $\bar{V^{r}}$ is the average value of the rth variable in cluster a.

### Spatial distribution differences of TVs

#### Overall pattern

**Fig 3:**
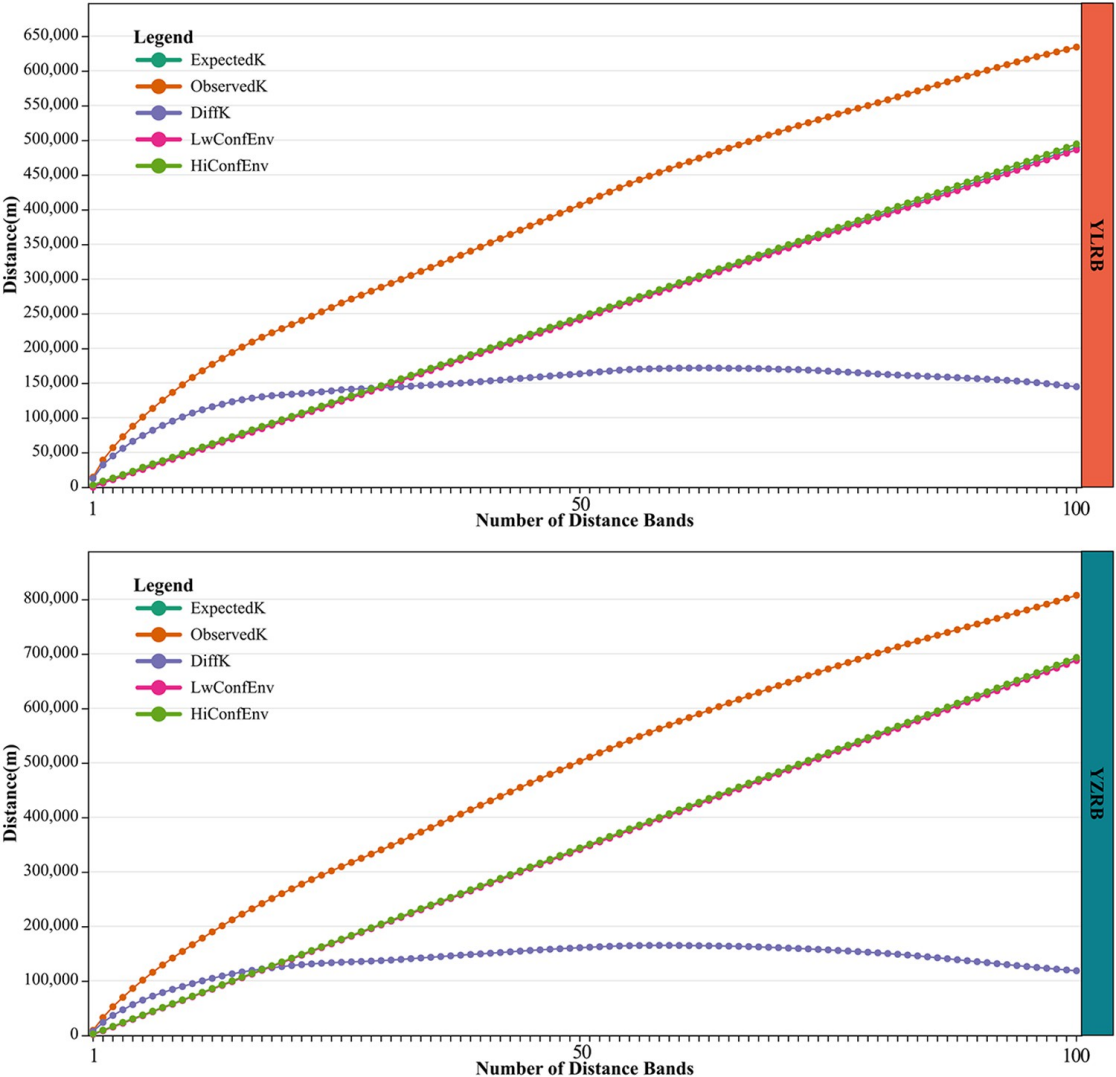
Multi-distance spatial cluster analysis of TVs in YLRB and YZRB.

Based on the space positions and space distances of TVs in the YLRB and YZRB, the distance scale increment was determined to conduct multi-distance spatial clustering analysis of the villages at various distance scales (Fig 3). The findings indicate that the ObservedK of TVs in both basins consistently exceeds the upper boundary of the HiConfEnv within the range of iterative distances. Moreover, the ObservedK curve consistently remains above the ExpectedK curve, revealing a pronounced clustering pattern of TVs within the iterative distance range.

**Fig 4:**
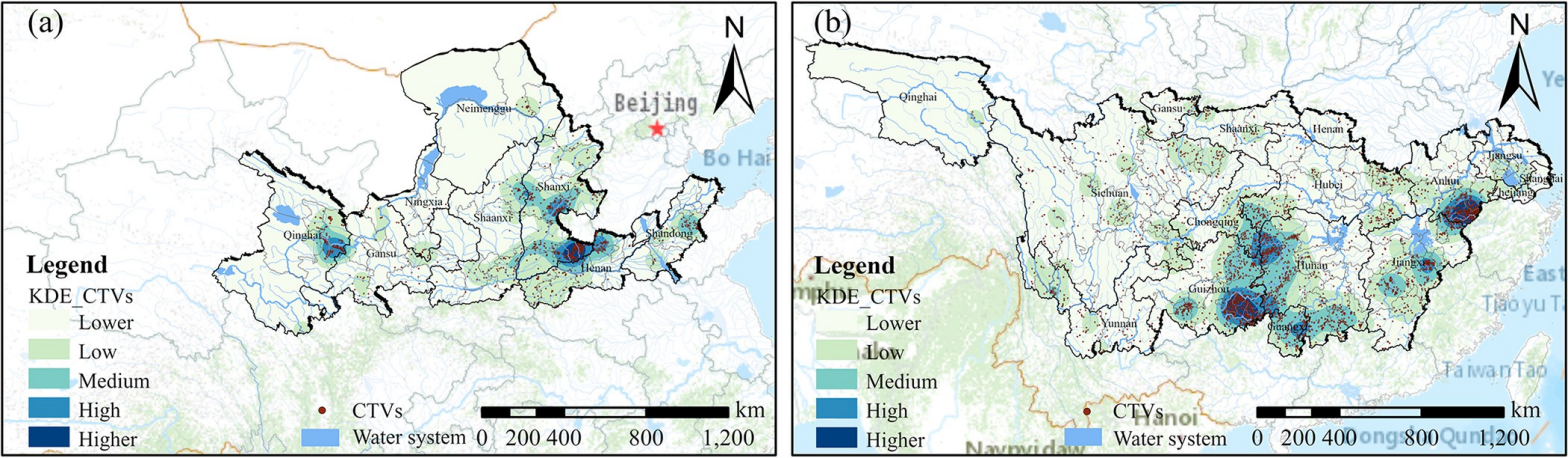
Kernel density analysis results of TVs. (a) YLRB. (b) YZRB. The maps were generated by ArcGIS 10.6 and were for illustrative purposes only.

Within the YLRB, the overall distribution of TVs exhibits a distinct pattern characterized by "one major center and two sub-centers" (Fig 4). The major center is situated where the provinces of Henan, Shanxi, and Hebei converge, while the two sub-centers are positioned along the line connecting Xining and Lanzhou, as well as in the vicinity of Taiyuan and its surrounding areas. In the YZRB, the distribution of TVs demonstrates a similar pattern with "two major centers and three sub-centers." One major center is situated in the southeastern part of Guizhou, near the border with Hunan, while the other major center is found in the southern part of Anhui, near the border with Jiangxi. Additionally, the three sub-centers are observed at the junction of Hunan, Guizhou, and Chongqing, in the southern region of Hunan, and in the central part of Jiangxi.

### Local autocorrelation

**Fig 5:**
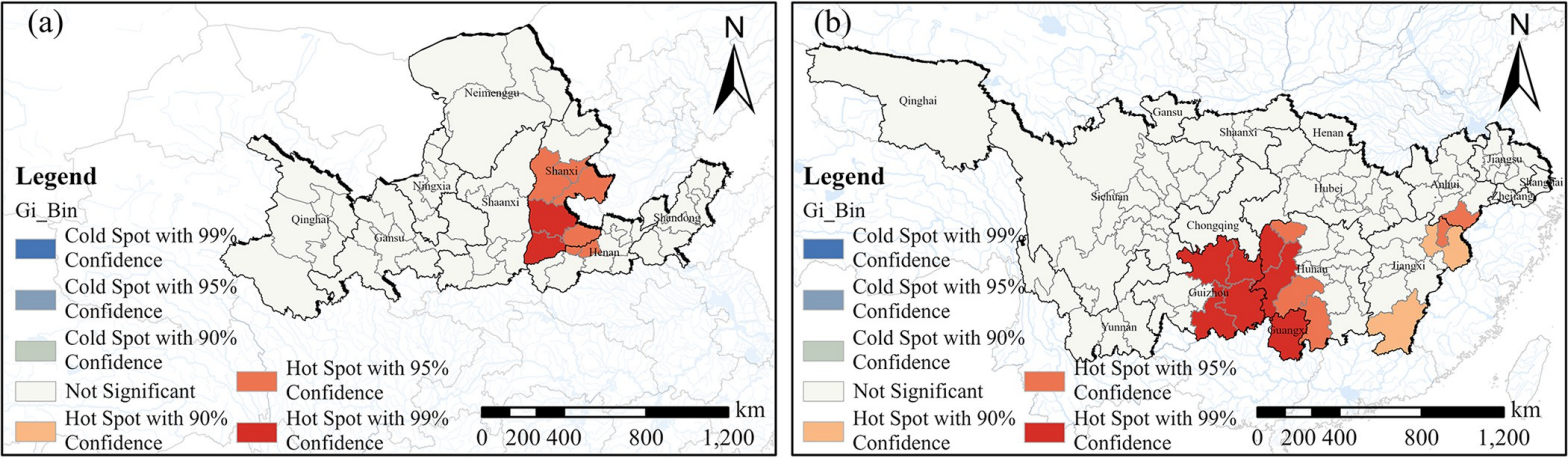
Spatial hotspot analysis of TVs. (a) YLRB. (b) YZRB. The maps were generated by ArcGIS 10.6 and were for illustrative purposes only.

The outcomes of the hotspot analysis performed at the county level reflect the geographical distribution traits of TVs in the two major river basins (Fig 5). At this refined scale, the spatial pattern of TVs distribution in the YLRB demonstrates a prominent "extreme hot-hot" pattern. The Yellow River’s middle reaches, primarily in Shaanxi province, are home to the majority of the areas with extreme hotspot values. Additionally, the hotspot areas exhibit a division, with one portion located in the middle reaches and another near the junction of the lower and middle reaches, covering areas within Shaanxi province and at the border between Shaanxi and Henan provinces. On the other hand, the space pattern of TVs in the YZRB at the county scale displays a discernible "extreme hot-hot-sub hot" pattern. The regions characterized by the extreme hotspot values are primarily situated in the Yangtze River’s upper and middle reaches, spanning across Guizhou, Hunan, and Guangxi provinces. Furthermore, the hotspot areas are observed in the Yangtze River’s middle and lower reaches, distributed within Hunan province and at the boundary between Jiangxi and Anhui provinces. Notably, areas with sub-hot spots are identified near the Yangtze River’s middle reaches, specifically within Jiangxi province. These findings shed light on the varying degrees of concentration and dispersion of TVs within different regions at the county level.

## Differences in driving factors

### Natural environment

#### Elevation and relief

**Fig 6:**
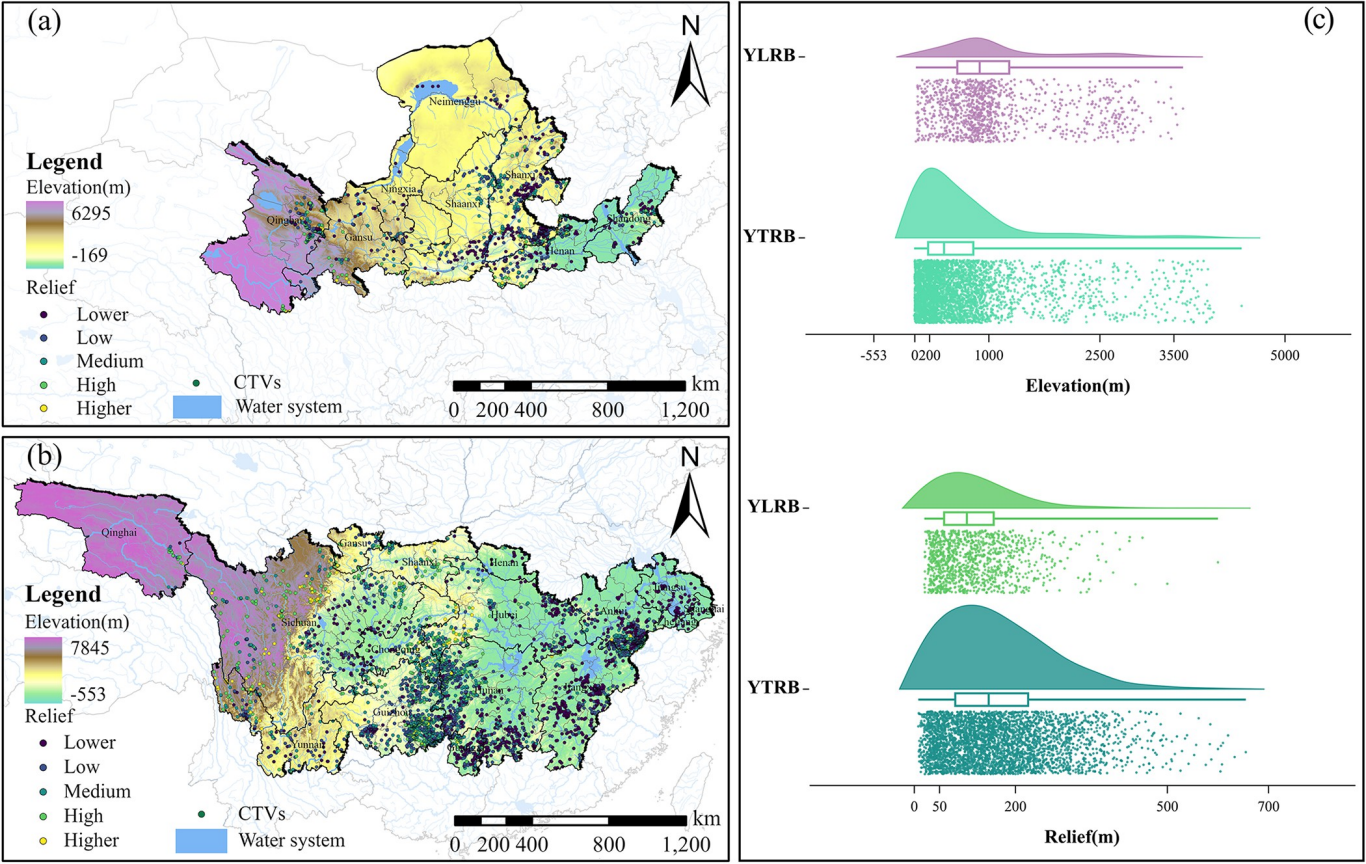
Analysis of the elevation and relief of TVs. (a) ELE and RE distribution of TVs in YLRB. (b) ELE and RE distribution of TVs in YZRB. (c) Count of TVs in each range of ELE and RE. The maps were generated by ArcGIS 10.6 and were for illustrative purposes only.

Within the YLRB, the highest number of TVs is observed in the 201–1000 meters elevation range, comprising 625 villages. TVs are predominantly concentrated in the mid to low-elevation zones, surpassing the count in areas with elevations exceeding 1500 meters. As elevation surpasses 1000 meters, the number of TVs distributions decreases with increasing elevation. Beyond an elevation of 3500 meters, only three TVs are found, and no TVs are present in regions exceeding 5000 meters in elevation. Regarding relief, 99.8% of TVs are situated in areas with relief below 500 meters, accounting for 1127 villages. Only two villages are located in the relief range of 500–1000 meters. Within the YZRB, the highest number of TVs is found in the elevation range of 201–1000 meters, totaling 2113 villages. The number of TVs increases with elevation within the elevation range of -553 meters to 200 meters. However, beyond an elevation of 200 meters, the number of TVs gradually decreases with increasing elevation, and no TVs are found above 5000 meters in elevation. As for relief, 99.3% of TVs are distributed in areas with relief below 500 meters, comprising 3363 villages. There are 27 villages in the relief range of 500–1000 meters. Overall, TVs are primarily situated in regions with lower elevations and relatively flat terrain (Fig 6).

#### Climate zones and water system

**Fig 7:**
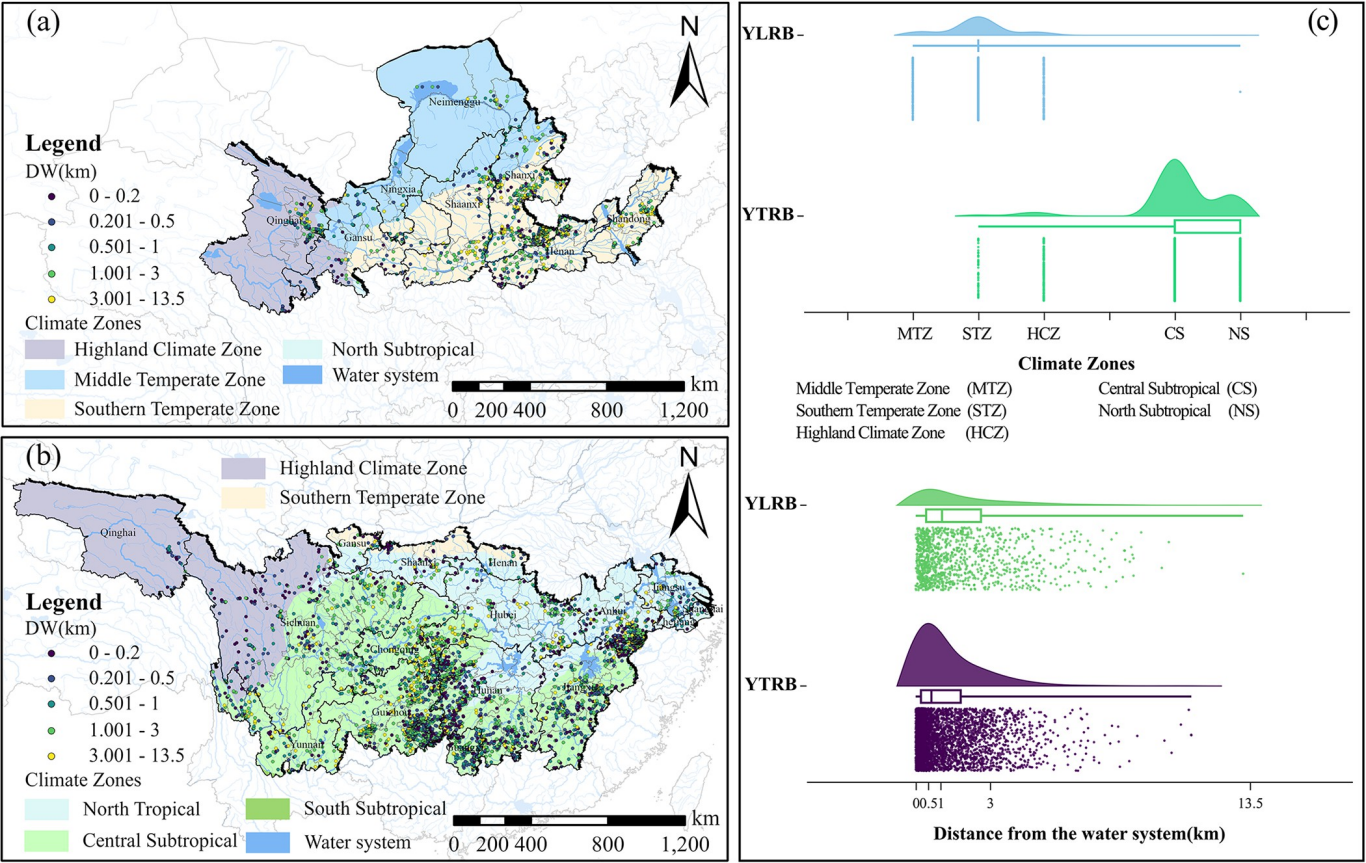
Analysis of the space configuration of TVs in relation to climate zone and water systems. (a) CZ and DW distribution of CTVs in YLRB. (b) CZ and DW distribution of CTVs in YZRB. (c) Count of CTVs in each range of CZ and DW. The maps were generated by ArcGIS 10.6 and were for illustrative purposes only.

The selection of village sites is closely related to climate and water resources (Fig 7). Within the YLRB, in terms of climate, the majority of TVs are distributed in the Southern Temperate Zone, totaling 823 villages, comprising 72.9% of the overall. The distribution of TVs in the Middle Temperate Zone and Highland Climate Zone is roughly equal, with only one in the North Subtropical Zone. Regarding proximity to water systems, most TVs are located within a distance of 0-3km, representing 78.7% of the total. A smaller number of TVs are situated at distances of 3–13.5km from water systems. Within the YZRB, in terms of climate, the majority of TVs are distributed in the Central Subtropical and North Subtropical zones, accounting for 94.4% of the total. A small portion of TVs are found in the Southern Temperate Zone and Highland Climate Zone. Similarly, in terms of proximity to water systems, most TVs are within a distance of 0-3km, representing 89.2% of the total, while the remaining 10.8% of TVs are located at distances of 3–13.5km from water systems. Overall, regions with relatively extreme and harsh climates tend to have fewer TVs, whereas areas near water systems are more conducive to developing TVs. However, to avoid the impact of water hazards, TVs are generally situated at a certain distance from water systems. Regions with pleasant climates and abundant water resources provide favorable conditions for agricultural production and human habitation, leading to a higher number of distributed TVs.

### Social culture

#### National intangible cultural heritages and national intangible cultural heritage inheritors

**Fig 8:**
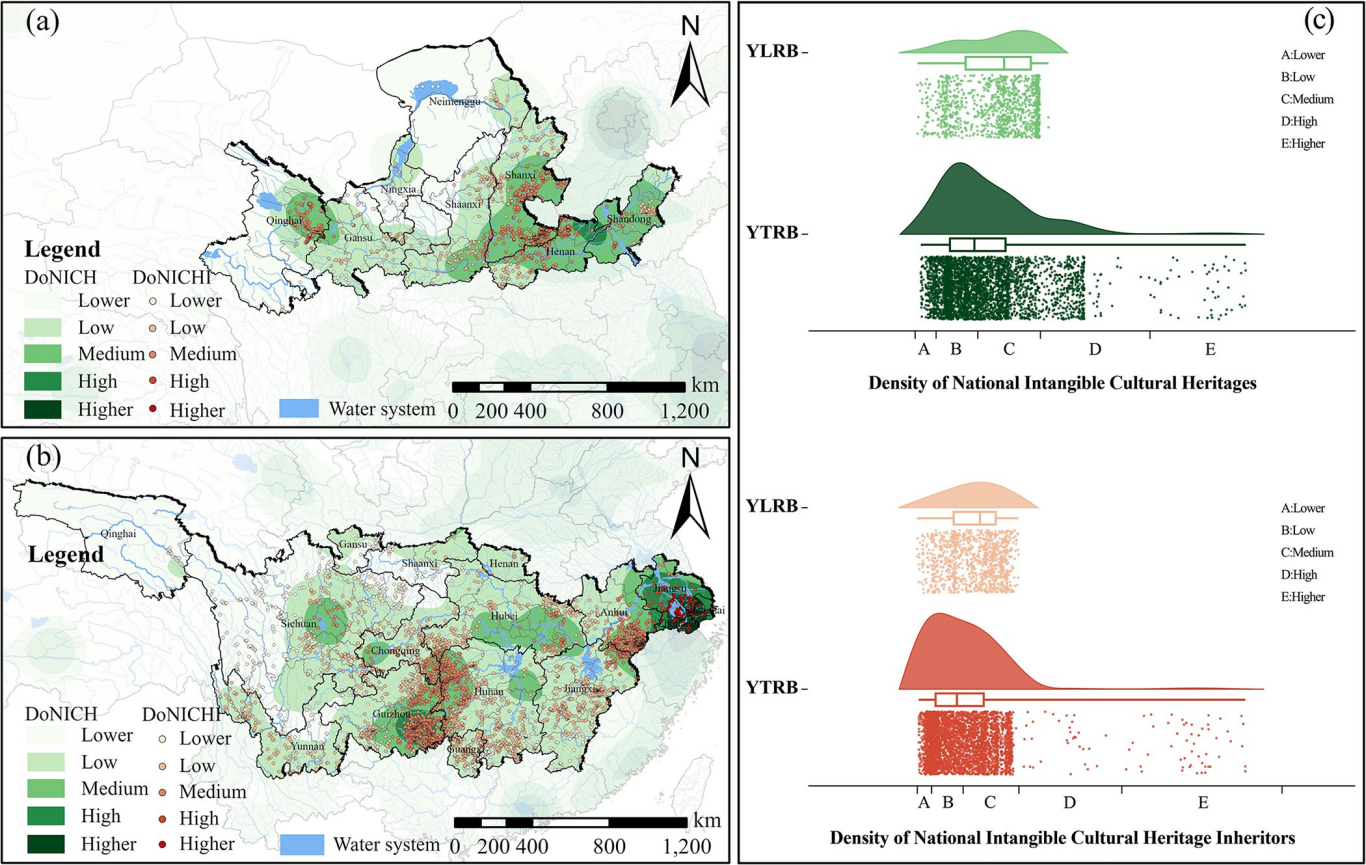
Analysis of the space configuration of TVs in relation to NICH and NICHI. (a) NICH and NICHI distribution of CTVs in YLRB. (b) NICH and NICHI distribution of CTVs in YZRB. (c) Count of CTVs in each range of NICH and NICHI. The maps were generated by ArcGIS 10.6 and were for illustrative purposes only.

Based on the collected data on National Intangible Cultural Heritages and National Intangible Cultural Heritage Inheritors, kernel density analysis was conducted to extract the quantity of TVs at different density levels in the YLRB and YZRB (Fig 8). Within the YLRB, as the Density of National Intangible Cultural Heritage Inheritors (DoNICH) increases, the quantity of distributed TVs initially increases, then decreases, and subsequently reaches a new peak before gradually declining. There are two peak points: one in the low Density of National Intangible Cultural Heritages (DoNICH) area and another in the moderate-density area. On the other hand, the quantity of TVs shows an increasing trend followed by a decrease as the DoNICHI increases. Within the YZRB, the distribution of TVs follows an inverted U-shaped pattern in relation to the DoNICH, with the highest quantity of TVs found in the low DoNICH area. The highest quantity of TVs is observed in the moderate DoNICHI area. The graph shows that areas with a concentrated distribution of TVs tend to have similar densities of intangible cultural heritage. In the YLRB, most TVs are distributed in the moderate DoNICH area. In contrast, in the YZRB, most TVs are distributed in the low-density and moderate-density areas of intangible cultural heritage. The inverse U-shaped association between the DoNICHI and the quantity of TVs also indicates that the areas with a concentrated distribution are not necessarily the same areas where intangible cultural heritage inheritors are concentrated.

**Fig 9:**
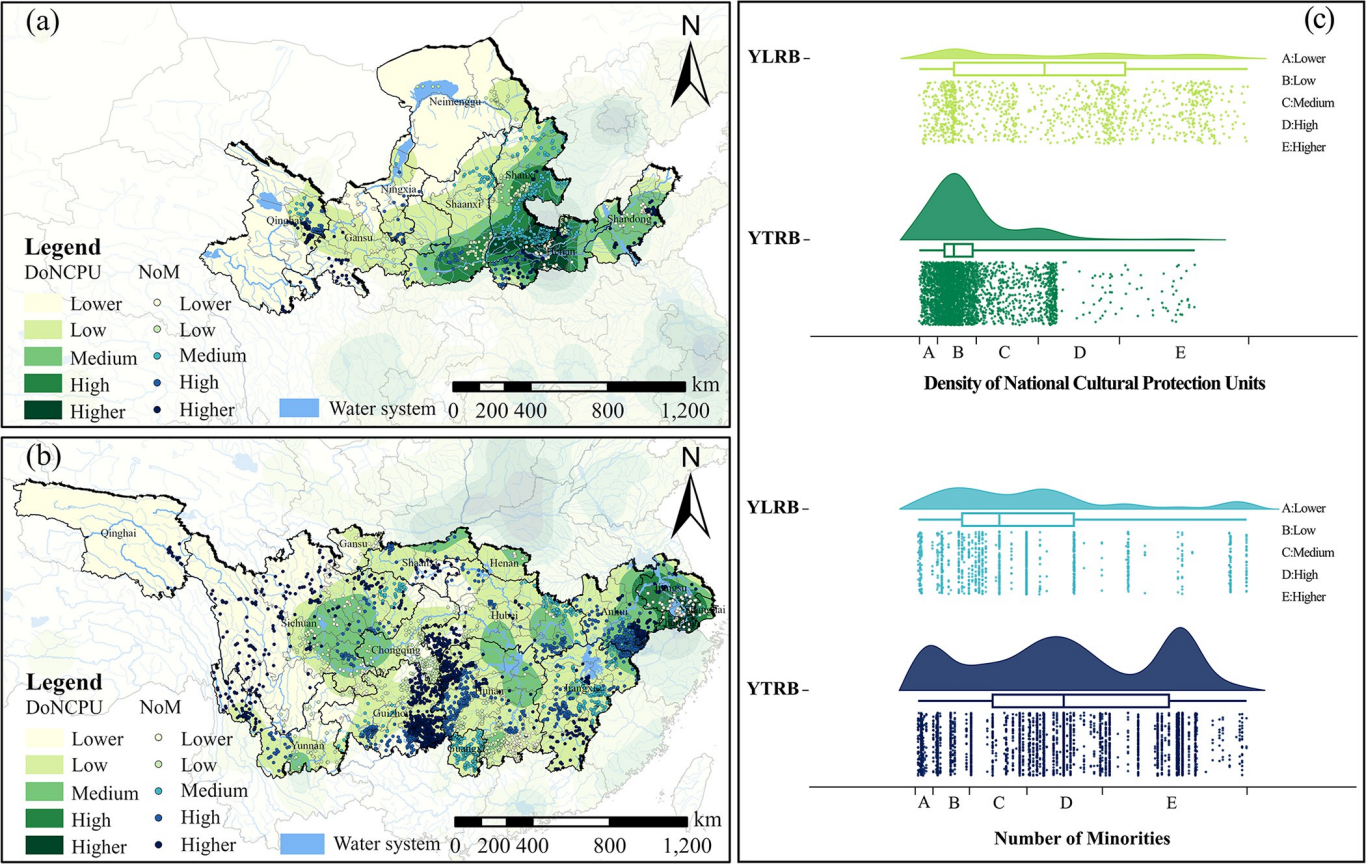
Analysis of the space configuration of TVs in relation to DoNCPU and NoM. (a) DoNCPU and NoM distribution of TVs in YLRB. (b) DoNCPU and NoM distribution of TVs in YZRB. (c) Count of TVs in each range of DoNCPU and NoM. The maps were generated by ArcGIS 10.6 and were for illustrative purposes only.

**National cultural protection units** and minorities. Within the YLRB, the quantity of TVs shows tiny variations with the fluctuation of National Cultural Protection Units density. There is little difference in the quantity of TVs distributed in different density areas. The relationship between the number of minority ethnic groups and the quantity of TVs follows a wave-like curve with minor fluctuations. Regions with more minority ethnic groups tend to have more distributed TVs, reaching 335. Within the YZRB, the highest quantity of TVs is found in the low-density area of National Cultural Protection Units, totaling 2369 and accounting for 64.2% of the total. Similarly, the relationship between the number of minority ethnic groups and the quantity of TVs also exhibits a wave-like curve but with more significant fluctuations than the YLRB. There are three peak points, and regions with more minority ethnic groups have more distributed TVs, reaching 2462 and accounting for 66.7% of the total (Fig 9).

#### Genealogies and language zones

**Fig 10:**
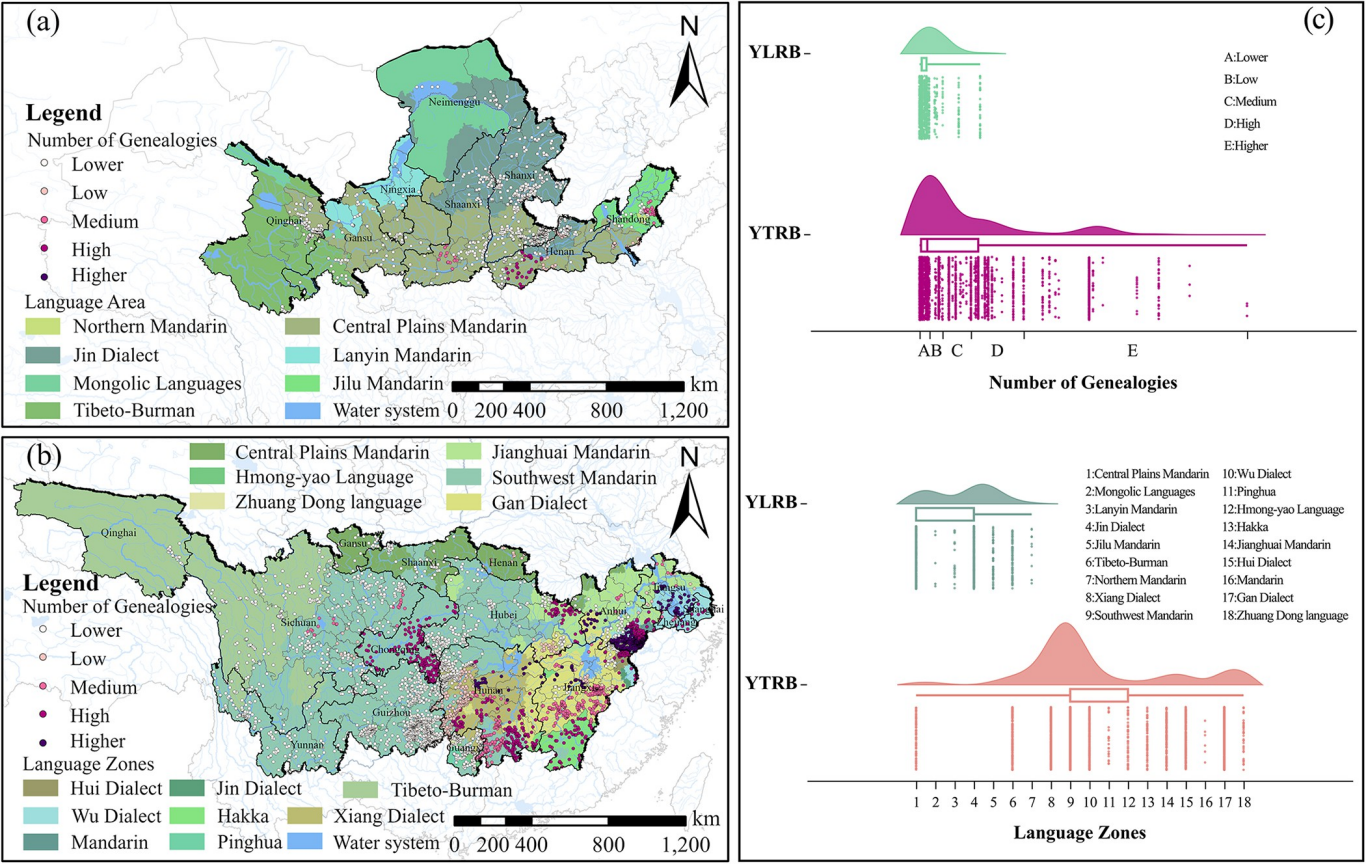
Analysis of the space configuration of TVs in relation to NoG and LZ. (a) NoG and LZ distribution of TVs in YLRB. (b) NoG and LZ distribution of TVs in YZRB. (c) Count of TVs in each range of NoG and LZ. The maps were generated by ArcGIS 10.6 and were for illustrative purposes only.

Based on the data from genealogical records and language regions at the prefectural scale, the distribution quantity of TVs was extracted in different layers (Fig 10). Within the YLRB, TVs initially peak as the number of genealogical records increases and then steadily decreases. The peak region corresponds to areas with fewer genealogical records, and spatially, TVs with a lower number of genealogical records are relatively densely distributed. There are seven language regions distributed within the YLRB, with a larger quantity of TVs found in Central Plains Mandarin and Jin Dialect. Within the YZRB, the quantity of TVs initially reaches a more prominent peak as the number of genealogical records increases, and then a smaller peak appears. 50% of TVs are concentrated between regions with a small to moderate number of genealogical records. More language regions, totaling 14 characterize the YZRB. The regions encompassing Southwest Mandarin, Xiang Dialect, Wu Dialect, Pinghua, and Hmong-yao Language have more distributed TVs, with over 50% of TVs located within these areas.

#### National A-class tourist attractions and national parks of China

**Fig 11:**
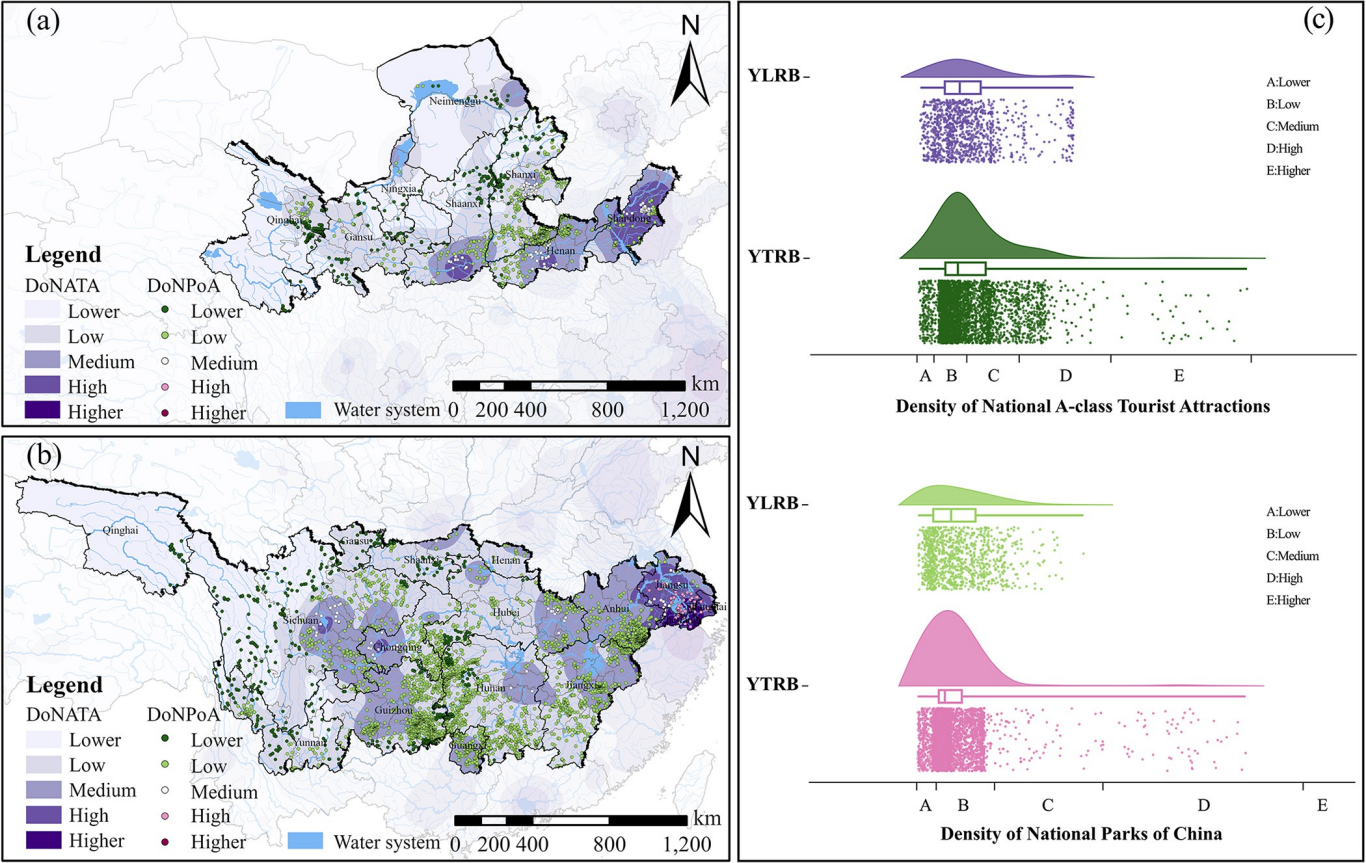
Analysis of the space configuration of TVs in relation to NoNATA and DoNPoA. (a) NoNATA and DoNPoA distribution of TVs in YLRB. (b) NoNATA and DoNPoA distribution of TVs in YZRB. (c) Count of TVs in each range of NoNATA and DoNPoA. The maps were generated by ArcGIS 10.6 and were for illustrative purposes only.

Based on the data of National A-class Tourist Attractions and National Parks of China, the analysis results using the same method mentioned above (Fig 11). Within the YLRB, the distribution of TVs initially increases and then decreases as the Density of National A-class Tourist Attractions (DoNATA) and Density of National Parks of China (DoNPoA) increase. From the figure, it can be observed that there are three regions with higher DoNATA and DoNPoA: the urban clusters centered around Xi’an, Zhengzhou, and Jinan. These regions have a higher level of urbanization, resulting in a relatively dispersed distribution of TVs within these areas. Within the YZRB, the distribution of TVs follows a pattern of initially increasing and then decreasing with the DoNATA and DoNPoA. When the DoNATA and DoNPoA are low, there is the highest quantity of TVs distributions. From the figure, it can be seen that the region with the highest DoNATA and DoNPoA is the Shanghai urban cluster. This region has a higher level of development and a high degree of land use intensity, resulting in a lower quantity of TVs distributions.

### Space economy

#### Transportation, urbanization and population

**Fig 12:**
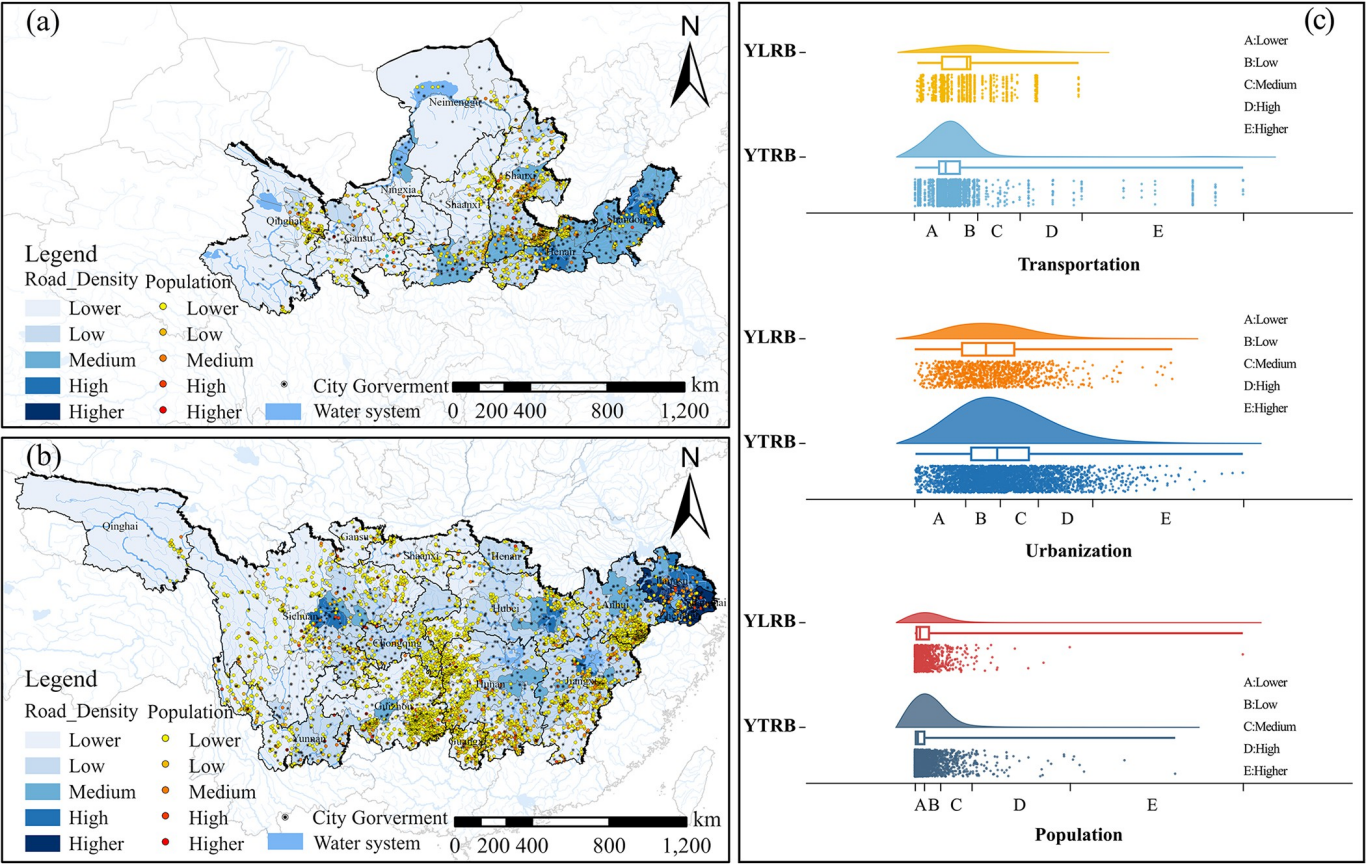
Analysis of the space configuration of CTVs in relation to Transportation (TRA), Urbanization (URB) and Population (POP). (a) TRA, URB and POP distribution of TVs in YLRB. (b) TRA, URB and POP distribution of TVs in YZRB. (c) Count of TVs in each range of TRA, URB and POP. The maps were generated by ArcGIS 10.6 and were for illustrative purposes only.

Within the YLRB, most TVs are concentrated in areas with low road network density, totaling 891 villages, accounting for 78.9%. As the scale of urbanization increases, the number of TVs initially increases and then decreases, with the peak occurring in areas with lower levels of urbanization. In terms of population density, the number of TVs initially increases and decreases as population density gradually increases. Within the YZRB, the highest number of TVs is found in areas with low road network density, and most TVs are distributed in regions with moderate to low levels of urbanization. Similar to the YLRB, most TVs are distributed in areas with lower population density (Fig 12).

#### Tourism income and rural residents’ per capita disposable income

**Fig 13:**
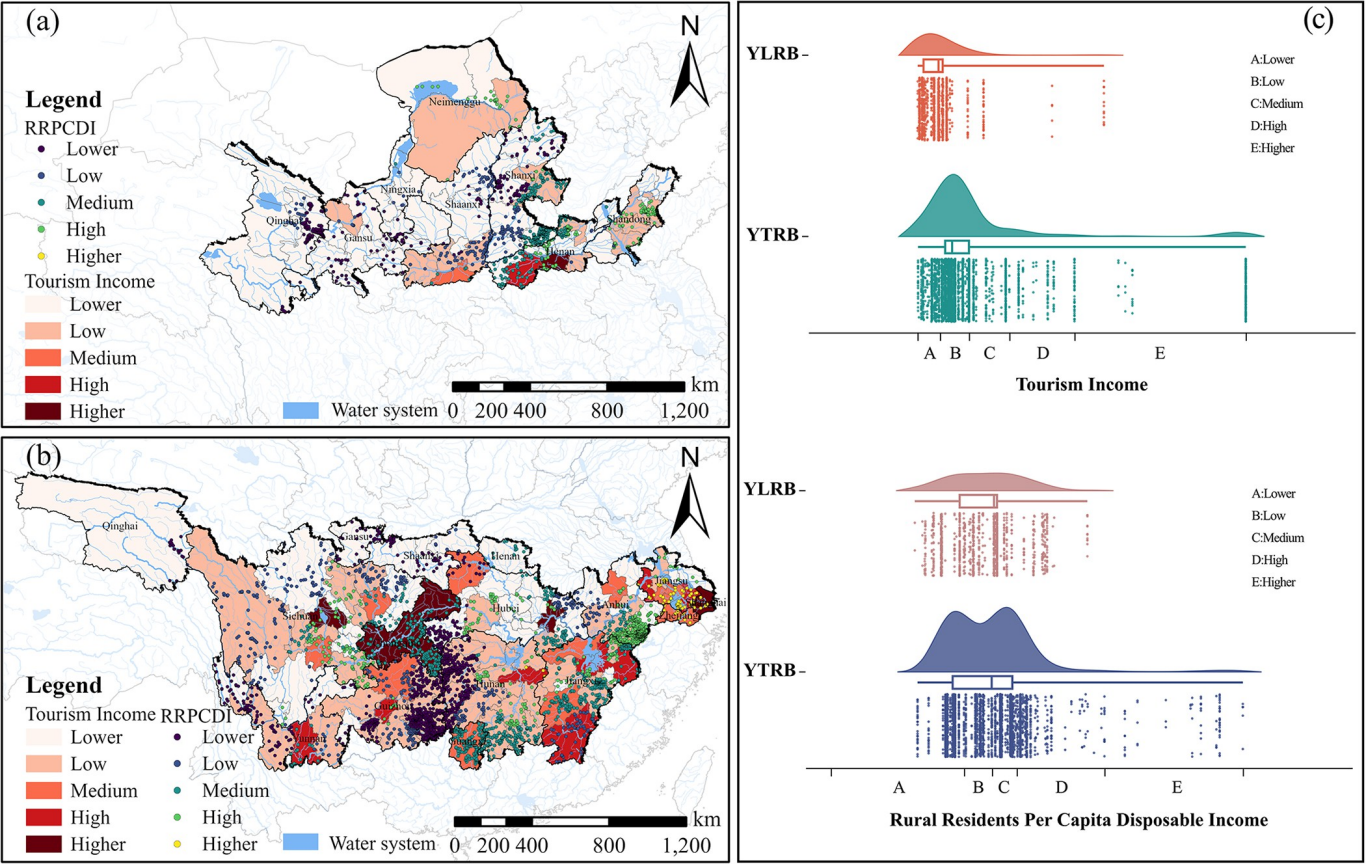
Analysis of the space configuration of TVs in relation to Tourism Income (TI) and Rural Residents Per Capita Disposable Income (RRPCDI). (a) TI and RRPCDI distribution of TVs in YLRB. (b) TI and RRPCDI distribution of TVs in YZRB. (c) Count of TVs in each range of TI and RRPCDI. The maps were generated by ArcGIS 10.6 and were for illustrative purposes only.

Within the YLRB, the majority of TVs are concentrated in areas with lower tourism income. As tourism income increase, the number of TVs initially increases and then decreases. Over 50% of TVs are distributed in lower or moderate rural residents’s per capita disposable income areas. In the YZRB, the number of TVs distributions follows a similar pattern, increasing and decreasing as tourism income increase. The peak occurs in areas with lower tourism income. Regarding rural residents’s per capita disposable income, the distribution of TVs shows two peaks as rural residents’s per capita disposable income increases. When rural residents’s per capita disposable income is lower or moderate, many TVs are found (Fig 13).

### Comprehensive impact factor analysis

#### Optimal discretization of continuous variables

**Fig 14:**
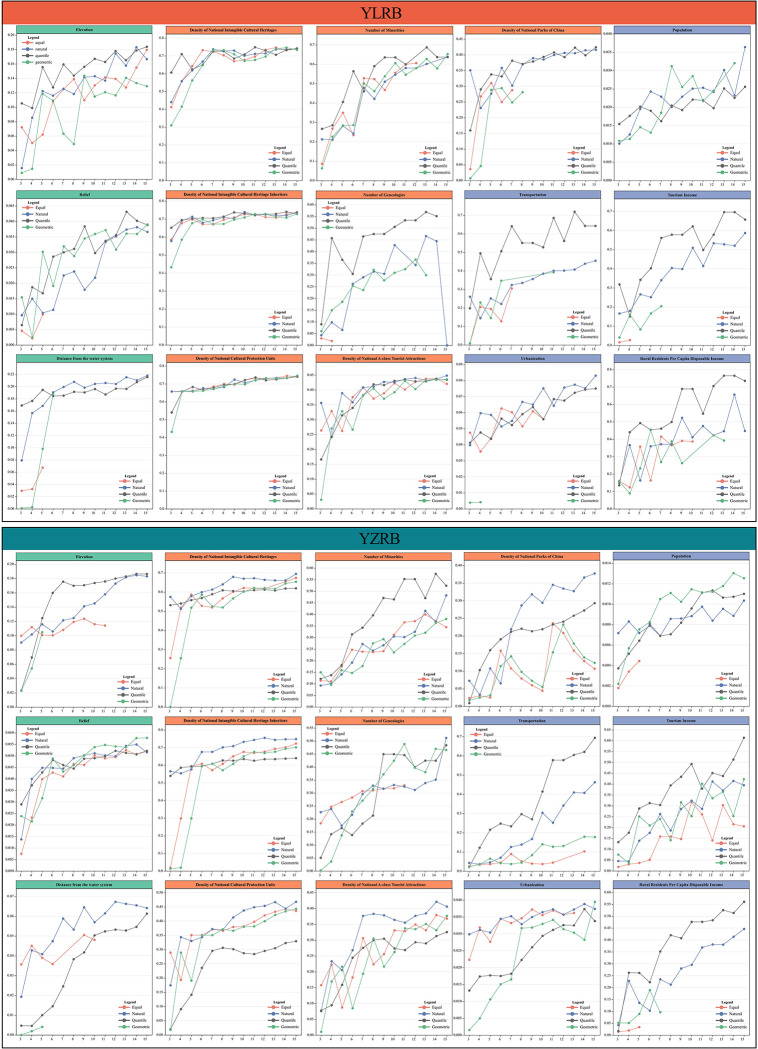
The best discretization results of each driving factor.

Utilizing the OPbGD approach, the study obtained the optimal discretization outcomes for continuous driving factors in YLRB and YZRB, encompassing ELE, RE, DW, DoNICH, DoNICHI, DoNCPU, NoM, NoG, DoNATA, DoNPoA, TRA, URB, POP, TI, and RRPCDI (Fig 14). The study employed four discretization methods, namely Equal, Natural, Quantile, and Geometric, while also computing the corresponding q-values to discretize the diverse categories of continuous driving factors. The optimal discretization scheme, based on the highest q-value, was selected to evaluate each driving factor’s influence on the spatial distribution of TVs, facilitating subsequent exploratory analysis. Table 2 provides the optimal classification results for each continuous variable category in YLRB and YZRB.

**Table 2:** Optimal discretization results for continuous drivers.

Continuous variable	YLRB		YZRB	
	Discretization method	Optimal classification	Discretization method	Optimal classification
ELE	Quantile	15	Quantile	14
RE	Quantile	13	Geometric	15
DW	Natural	15	Natural	14
DoNICH	Natural	7	Natural	15
DoNICHI	Quantile	14	Natural	12
DoNCPU	Geometric	15	Natural	14
NoM	Quantile	13	Quantile	14
NoG	Quantile	13	Geometric	11
DoNATA	Natural	15	Natural	15
DoNPoA	Quantile	15	Natural	15
TRA	Quantile	13	Quantile	15
URB	Natural	15	Natural	11
POP	Natural	14	Geometric	14
TI,	Quantile	13	Quantile	15
RRPCDI	Quantile	13	Quantile	15

#### Single factor detection

**Fig 15:**
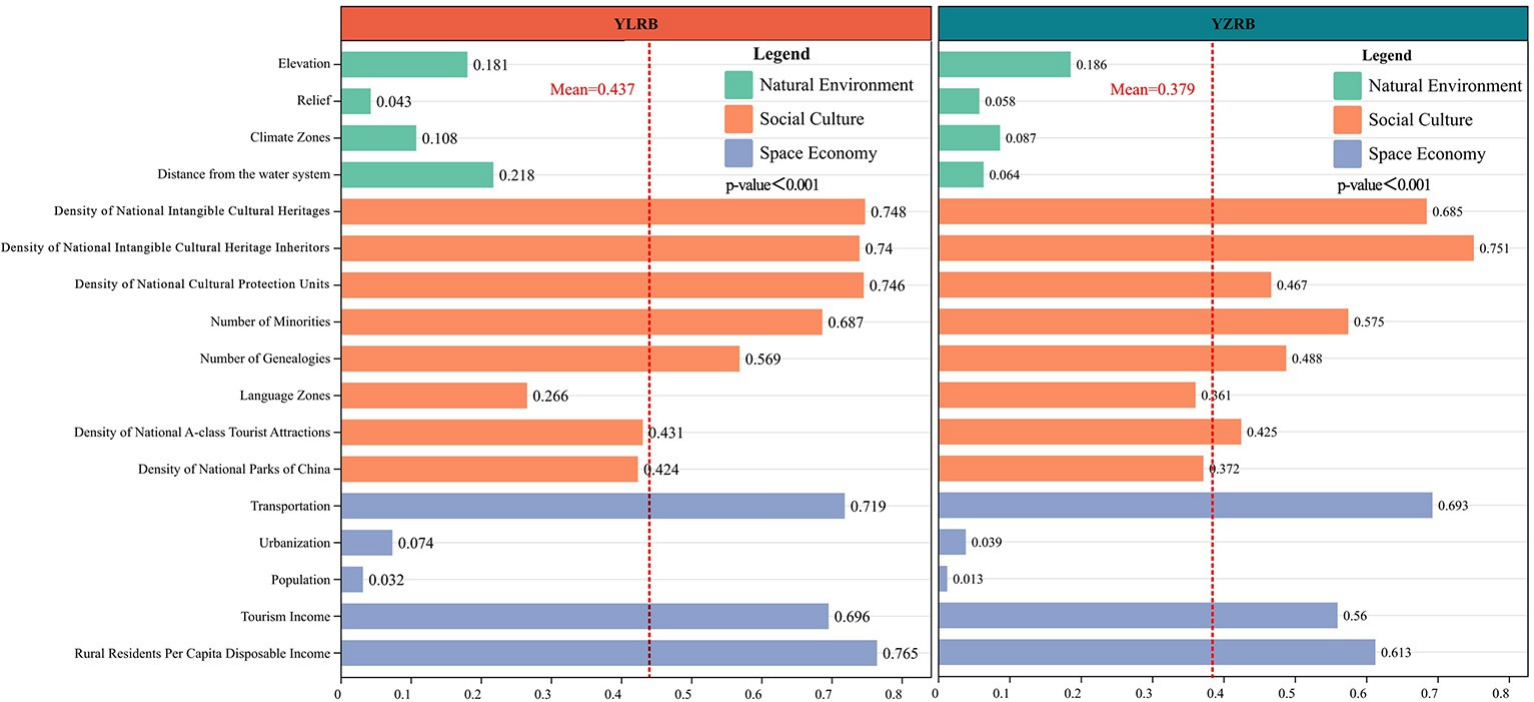
Comparison of the impact of each driving factor between YLRB and YZRB.

The influence of each driving factor on the space pattern of TVs in the YLRB and YZRB is represented by their respective q-values (Fig 15). Regarding the natural environment, DW exhibits the highest impact on the space pattern of TVs in the YLRB. In contrast, its influence on the pattern in the YZRB is merely 0.064, indicating the crucial role of the water system in shaping the pattern of TVs, specifically in the YLRB. In terms of social culture factors, DoNICH and DoNCPU demonstrate the most decisive influence on the TVs’ pattern in the YLRB, while DoNICHI notably affects the space configuration of TVs in the YZRB, with DoNCPU ranking fifth with a modest impact value of 0.467. Concerning space economy, TRA, TI, and RRPCDI exhibit significant impacts on the space configuration of TVs in both the YLRB and YZRB. Among them, RRPCDI emerges as the driving factor with the most decisive influence on the space configuration of TVs in the YLRB. In contrast, TRA plays the most prominent role in shaping the pattern in the YZRB. Overall, social culture factors play pivotal roles in determining the space configuration of TVs in both basins, whereas the impact of natural environment factors appears less pronounced. Except for URB and POP, among the space economy factors, which demonstrate insignificant influences, TRA, TI, and RRPCDI exert substantial effects.

#### Factors interplay detection

**Fig 16:**
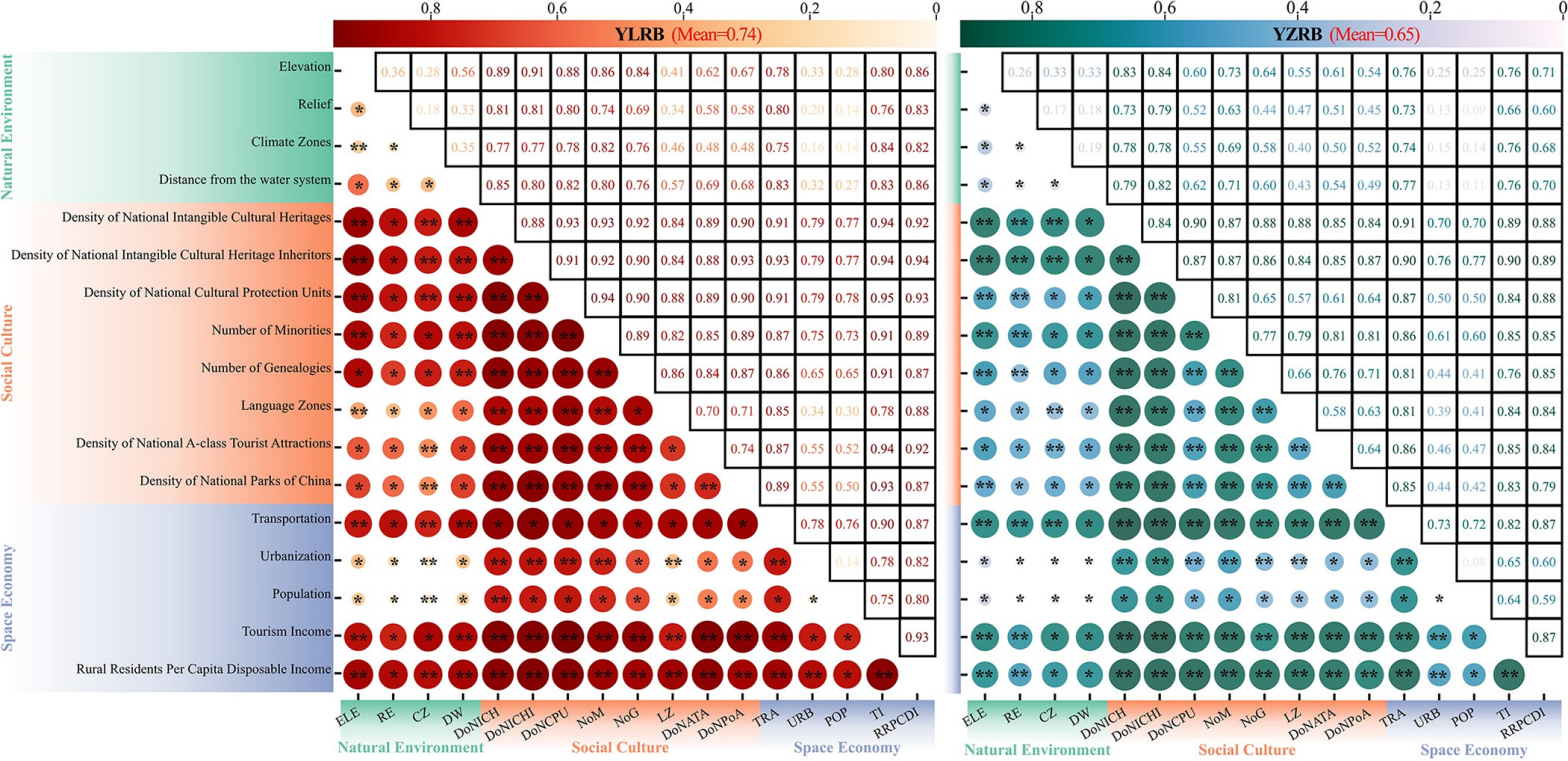
Comparison of interaction effects of driving factors between YLRB and YZRB.

According to the interaction detection results among the drivers (Fig 16), within the YLRB in China, the interaction between the Density of National Cultural Protection Units and Tourism Income (DoNCPU∩TI) exhibits the most potent interaction effect with a q-value of 0.9544. In contrast, within the YZRB, the interaction between the Density of National Intangible Cultural Heritage Inheritors and Transportation (DoNICHI∩TRA) demonstrates the most prominent interaction effect with a q-value of 0.9099. Interestingly, it is observed that in the YLRB, the interaction types with Density of National Intangible Cultural Heritages are consistently classified as "Enhance, bi-", while the interaction types with Relief are categorized as "Enhance, nonlinear." In the YZRB, the interaction types with Distance from the water system and Population are identified as "Enhance, nonlinear." Furthermore, Table 3 presents the interactions among the top five driving factors based on their q-values.

**Table 3:** Interactions among the top five drivers of q-value.

YLRB			YZRB		
Interactive variable	Interaction type	q-value	Interactive variable	Interaction type	q-value
DoNCPU∩TI	Enhance, bi-	0.9544	DoNICHI∩TRA	Enhance, bi-	0.9099
DoNCPU∩NoM	Enhance, bi-	0.943	DoNICHI∩ RRPCDI	Enhance, bi-	0.9081
DoNICH∩TI	Enhance, bi-	0.9381	DoNICHI∩TI	Enhance, bi-	0.8955
DoNATA∩TI	Enhance, bi-	0.938	DoNICH∩TRA	Enhance, bi-	0.8944
DoNICHI∩TI	Enhance, bi-	0.9375	DoNICHI∩DoNCPU	Enhance, bi-	0.8916

### Geographically similarity

**Fig 17:**
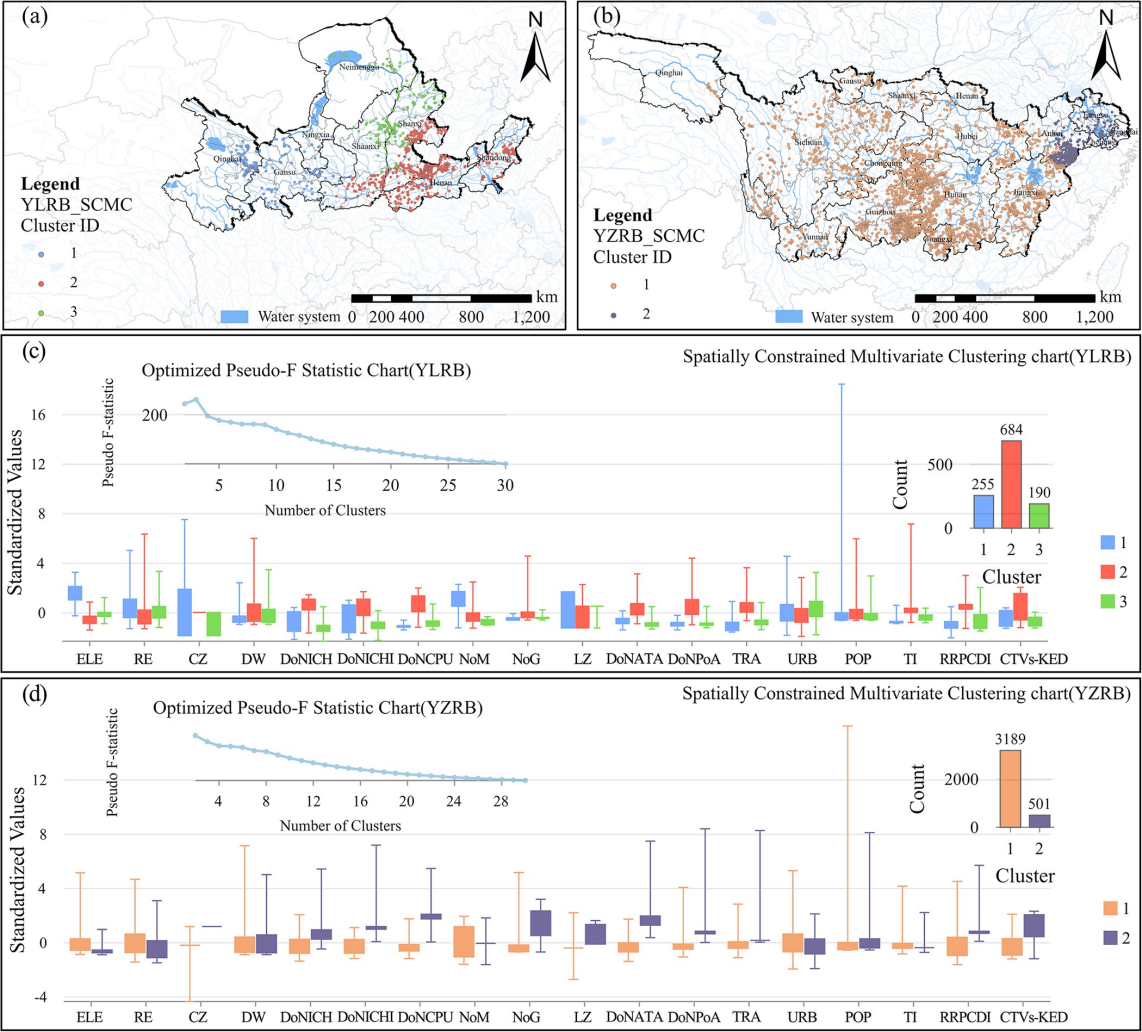
Spatially constrained multivariate clustering of TVs in YLRB and YZRB. The maps were generated by ArcGIS 10.6 and were for illustrative purposes only.

Building upon the 17 driving factors mentioned earlier and the kernel density analysis of TVs, the SCMC analysis method was employed to investigate the geographical similarities and clustering of TVs in the YLRB and YZRB. The clustering approach with the highest pseudo-F-statistic was selected as the optimal classification (Fig 17). In the YLRB, the TVs were grouped into three major clusters. The majority of the 255 TVs in Cluster 1 were situated in the upper Yellow River, specifically in Qinghai, Gansu, and Ningxia. Cluster 2 consisted of the largest number of TVs, totaling 684, and was primarily distributed in the southern regions of Shaanxi and Shanxi and Henan and Shandong in the Yellow River’s lower reaches. Cluster 3 was primarily concentrated in Neimenggu, northern Shaanxi, and western Shanxi, comprising only 190 TVs. Interestingly, the 3,690 TVs in the YZRB were classified into only two major clusters. Cluster 1, the largest in quantity, included 3,189 TVs primarily situated in the Yangtze River’s upper and middle-lower reaches, covering most of the basin area. Cluster 2 was mainly located in the estuary region of the Yangtze River, encompassing economically developed areas such as the southeastern part of Anhui, southern Jiangsu, eastern Zhejiang, and Shanghai. However, this cluster comprised only 501 TVs.

## Discussion and conclusion

### Discussion

#### The driving factors of the spatial differentiation of traditional villages

In summary, the primary influence on the spatial distribution of TVs is attributed to social and cultural factors, followed by economic factors, with natural environmental factors having a relatively lesser impact. It is noteworthy that the spatial evolution of TVs is driven by a complex interplay of these three dimensions: natural, social, and economic [19]. During the initial phases of settlement formation, the natural environment significantly influences the choice of settlement locations [51], often favoring areas of lower elevation and proximity to water sources for TVs. However, as societies progress, village development encounters various external challenges, including natural disasters, territorial expansion, cultural assimilation, urban sprawl, and internal risks such as disruptions in cultural heritage and internal divisions. Consequently, in the evolutionary stages of village development, the role of social and cultural factors and spatial-economic factors becomes increasingly pivotal in shaping the spatial distribution of TVs. The preservation of these enduring settlements necessitates a certain level of economic support and, more critically, the stable transmission of culture.

Firstly, the q-value for DW in the YLRB is 0.218, whereas, in the YZRB, it is 0.064. This suggests a more pronounced impact of the water system on the space pattern of TVs in the YLRB, attributable to the uneven overall distribution and distinct local disparities in the water system within the region. Secondly, the YZRB exhibits more national cultural protection units, a more uniform distribution, and a significant presence of ethnic minorities, leading to diverse linguistic regions. Consequently, significant differences in the q-values of DoNCPU and LZ exist between the YZRB and the YLRB. Furthermore, the substantial internal variations in tourism income and rural residents’ per capita disposable income among TVs in the YLRB contribute to markedly higher q-values for TI and RRPCDI than those observed in the YZRB.

#### Geographic similarities of traditional villages

TVs in the same region with a comparatively stable living environment will be influenced by a comparatively stable historical and cultural endowment. They will have similar folk traits, cultural environments, and development needs [28]. Moreover, the villages have a point-group structure in geographical space. The characteristics of settlements are derived from different geographical environments, a relationship based on commonality rather than individuality. Discerning clusters of TVs with analogous geographical features is crucial in facilitating their preservation and sustainable development.

#### Suggestions on the protection and development of traditional villages

Advocate for concentrated and interconnected preservation and development of TVs. Transitioning from the historical ’single-village preservation and development’ model to the ’concentrated and interconnected preservation and development’ approach, with TVs as nodes, connecting them systematically to optimize regional resources. This formation leads to resource scaling and diversified development, enabling a synergy among villages, thereby mitigating issues related to the homogeneity and singular development patterns observed in traditional village development.

Formulate distinct development strategies to promote tourism in the Yellow Rivers Basin and Yangtze Rivers Basin. Due to significant differences in the levels of tourism development, reflective of variations in natural environments, cultural resources, economic development, and tourism strategies, it is essential to adapt strategies accordingly. The upper reaches of the Yellow River and the middle-upper reaches of the Yangtze River enjoy superior natural conditions, abundant cultural resources, and higher economic development levels, resulting in relatively advanced tourism development. Conversely, the Yellow River’s middle-lower reaches, and the Yangtze River’s lower reaches exhibit relatively poorer natural conditions and lower economic development levels, leading to comparatively weaker tourism development. To address regional development disparities, it is crucial to formulate differentiated tourism development strategies tailored to each region’s unique characteristics and advantages. For instance, the Yellow River’s upper reaches and the Yangtze River’s middle-upper reaches can focus on enhancing tourism service quality. In contrast, the Yellow River’s middle-lower reaches and the Yangtze River’s lower reaches should prioritize infrastructure development, service capacity enhancement, and the promotion of distinctive tourism products and themes. It is imperative to strike a balance to prevent overdevelopment and avoid harm to the local environment and cultural resources.

Promote regional tourism. In regions with relatively uniform geographical features, cities can serve as focal points to develop regional tourism plans based on natural environments, social culture, spatial economics, and other resource factors. These plans should align with the preferences of tourists and attract visitors to explore within specific regional boundaries.

#### Limitations and potential research directions

Nonetheless, this study has several limitations that should be acknowledged. Firstly, in terms of selecting the geographical detector model, there are various models available such as the GOZH model [52], RGD model [53], and GHM model [54]. However, this study did not conduct a comparative analysis to determine the most suitable model for investigating the spatial heterogeneity of TVs. Secondly, the study did not delve into the specific ethnic groups and their distinct cultures within TVs. Furthermore, the research on the geographical similarity of TVs needs to be more in-depth. It can be discussed separately from the aspects of the natural environment, social culture, and spatial economy, or the degree of similarity can be evaluated. We also did not consider the relationship between the historical agricultural and pastoral ecotone and the distribution of traditional villages. These limitations highlight potential areas for future research endeavors.

## Conclusion

This study investigates the variations in spatial heterogeneity and geographical similarity among TVs in the YLRB and the YZRB. The main conclusions are as follows:

1. TVs generally exhibit clustering distribution patterns in both significant river basins. Within the YLRB, the distribution of TVs shows a pattern characterized by "one major center and two sub-centers." The primary center is at the intersection of Henan, Shanxi, and Hebei provinces. At the same time, the two sub-centers are situated along the line connecting Xining and Lanzhou and in the vicinity of Taiyuan. In the YZRB, the distribution of TVs demonstrates a pattern of "two major centers and three sub-centers." The first significant center is located at the southeastern border of Guizhou and Hunan provinces. In contrast, the second major center is situated at the southern border of the Anhui and Jiangxi provinces. The three sub-centers are found at the junction of Hunan, Guizhou, and Chongqing, in the southern part of Hunan and the central part of Jiangxi.
2. The OPbGD model is implemented to analyze the spatial heterogeneity of TVs in both basins, providing insights into the key influencing factors. Among the three major categories and seventeen driving factors considered in this study, the most influential factor influencting the space pattern of TVs in the YLRB is identified as RRPCDI, while for the YZRB, it is found to be DoNICHI. Notably, the impact of POP on the spatial heterogeneity of TVs in both basins is not statistically significant. Additionally, by examining the interactions between different factors, it is observed that the combined effects of pairwise factors further enhance the spatial heterogeneity of TVs. In the YLRB, the interaction between Density of National Cultural Protection Units and Tourism Income (DoNCPU∩TI) exhibits the highest level of influence on the space pattern of TVs, with a value of 0.9544. In the YZRB, the interaction between Density of National Intangible Cultural Heritage Inheritors and Transportation (DoNICHI∩TRA) shows the highest q-value of 0.9099.
3. The SCMC method is employed to classify the geographical similarity of TVs in the two basins, resulting in three distinct clusters for the TVs in the YLRB and two clusters for those in the YZRB.
